# Burning Issues/Hot Topics: The Charcot Foot

**DOI:** 10.3390/jcm15051993

**Published:** 2026-03-05

**Authors:** Victoria E. L. Milbourn, Ava Khoshnaghsh, Michael E. Edmonds, Nina L. Petrova

**Affiliations:** 1 Department of Rehabilitation Medicine, Amsterdam University Medical Centre, University of Amsterdam, 1105 AZ Amsterdam, The Netherlands; v.e.l.milbourn@amsterdamumc.nl; 2 Amsterdam Movement Sciences, Rehabilitation and Development, Amsterdam University Medical Centre, University of Amsterdam, 1105 AZ Amsterdam, The Netherlands; 3 Mike Edmonds Foot Unit, King’s College Hospital National Health Service Foundation Trust, London SE5 9RS, UK; michael.edmonds@nhs.net; 4 Guy’s, King’s and St Thomas’ School of Medicine, Faculty of Life Sciences and Medicine, King’s College London, London SE5 9RS, UK; ava.khoshnaghsh@kcl.ac.uk; 5 School of Cardiovascular and Metabolic Medicine and Sciences, Faculty of Life Sciences and Medicine, King’s College London, London SE1 7EH, UK

**Keywords:** Charcot neuro-osteoarthropathy, diabetic neuropathy, clinical management

## Abstract

Charcot neuro-osteoarthropathy remains one of the most serious complications of diabetic neuropathy, with the potential for rapid progression to deformity, ulceration and limb-threatening outcomes. The earliest signs and symptoms are often subtle and easily missed, yet early recognition provides a narrow window of opportunity to prevent irreversible structural change. Contemporary diagnostic pathways are underpinned by close clinical observation and correlation of bedside findings with detailed imaging analysis, enabling earlier identification of active disease before collapse occurs. This article provides a comprehensive overview of the Charcot foot, integrating clinical presentation, differential diagnosis and current approaches to investigation and management. It also highlights the challenges of monitoring disease activity over time, including remission and relapse, and the importance of timely specialist referral and multidisciplinary management. By reinforcing structured assessment and targeted intervention, this review aims to support consistent, evidence-informed care for people affected by Charcot neuro-osteoarthropathy.

## 1. Introduction

The Charcot foot in diabetes is an inflammatory process in persons with neuropathy, which results in injury to bones, joints, and soft tissues of the foot and ankle [1]. This soft tissue and bone injury may lead to loss of the architecture of the foot and ankle and result in long-term deformity due to fractures, dislocations, and fracture-dislocations [2]. The condition was named after the French neurologist Jean-Martin Charcot, who reported neuropathic bone and joint disease in tabes dorsalis in 1868 [3]. Later, Charcot joints have been described in other neuropathies, including leprosy, congenital sensory neuropathy, familial amyloid neuropathy, alcohol neuropathy and more recently in human immunodeficiency virus (HIV)-induced neuropathy.

William Reily Jordan was the first to link diabetes and Charcot neuro-osteoarthropathy (CNO) [4]. In 1936, he described “a rather typical, painless Charcot joint of the ankle” of a 56-year-old woman with diabetes of fourteen years’ duration. He concluded that on the background of negative tests for syphilis, the observed disturbances were most likely related to ‘a diabetic process of a neurologic trophic nature.’

Over the last few decades, there has been a marked increase in the recognition and reporting of CNO in diabetes [5]. Furthermore, with the global rise in the prevalence of diabetes, the prevalence of diabetes-related complications, including CNO, is also expected to rise. Thus, the overall objective of this manuscript is to raise awareness of CNO among the wider clinical community for improved recognition and timely referral for specialist management.

## 2. The Facts and a Call upon Early Recognition

### 2.1. Epidemiology of CNO in Diabetes

The true prevalence and incidence of CNO are not fully known. A recent regional audit carried out in seven hospital-based specialist services noted that the prevalence of CNO in the East Midlands region of England during a single month was 4.3 per 10,000 people with diabetes [6]. A Danish national patient register study reported that the prevalence of Charcot foot was 0.56% (1722 with Charcot foot of 309,557 people registered with an ICD-10 code of diabetes from 1995 to 2018). The incidence rate of Charcot foot was 7.4 per 10,000 person-years [7]. In the US population with diabetes, the estimated incidence of CNO is considered to be 27,602 per year and the prevalence to be 208,880 persons [8]. To put this in perspective, the authors reported that the incidence of CNO is higher than that of prostate, lung, kidney, and thyroid cancer and also higher than the incidence of multiple myeloma, soft tissue sarcoma, and primary bone sarcoma [8].

Some of these differences could be related to inconsistency in the nomenclature [9], which has now been standardised to CNO of the foot and ankle in people with diabetes mellitus, or Charcot foot [1]. A further explanation could be inconsistency in using established international disease codes. A study in Italy showed that three different international disease codes were used to record newly diagnosed cases of Charcot foot [10]. Thus, it is likely that CNO in diabetes is more common than reported [11]. This directs a need for large population-based studies using consistent nomenclature and coding system to confirm the true incidence and prevalence of CNO in people living with diabetes.

### 2.2. Why Is CNO Often Diagnosed Late?

It is now well recognised that diabetes is the leading cause of CNO. However, the condition is frequently underreported or misdiagnosed. Data from a recent systematic review underscored that the initial presentation of the Charcot foot is overlooked in up to 95% of cases, with a median diagnostic delay of approximately 87 days (95% CI: 10.5–162.1) [12,13]. Often, the condition is only suspected when patients have developed significant foot deformity. The latter sets off a chain of adverse events with a high risk of limb loss and death [14].

The reasons for this diagnostic delay are multifactorial [15]. Individual awareness and health professional awareness of the presenting signs and symptoms, together with geographical barriers in accessing care, seem to be contributing factors. Surveys among non-foot specialists in the USA and Denmark have indicated that almost 70% of service providers admitted either minimal or non-existent knowledge of CNO [16,17]. Also, healthcare professionals have expressed uncertainty about how to make the diagnosis, when to institute medical treatment or when to escalate care and refer to specialist treatment.

As well as poor recognition by healthcare professionals, the lack of urgency in seeking help by the person living with diabetes is a further contributing factor to delayed presentation and referral. The lack of interest in foot health, combined with the inability to detect warning signs due to neuropathy and uncertainty of what to look for and when to seek help, leads to a lack of urgency in people with diabetes [18]. These are often aggravated by physical, social and mental health factors, all of which account for delayed presentation. This calls upon an urgent need for wide recognition and improved awareness of the presenting signs and symptoms of CNO amongst healthcare professionals and people living with diabetes, as the outlook of the condition and its long-term consequences are poor.

### 2.3. The Impact of CNO on Society and Individuals

The socio-economic and personal impact of CNO in diabetes is immense. Both the management of the CNO and its associated complications place a considerable strain on healthcare systems, driving high rates of inpatient admissions, emergency department visits, outpatient reviews, and long-term follow-up with podiatry and orthotic departments [19]. Delayed recognition and misdiagnosis are adversely related to poor functional outcome, reduced quality of life and need for lifelong support [10,20,21,22]. Additionally, a recent longitudinal study has implicated social determinants of health (living alone or with one cohabitant, distance to clinic, living in a rural area, employment status and education) as further contributing factors to prolonged healing time in CNO [23]. In the longer term, up to 80% of Charcot feet develop foot ulcers, and the risk of major (above-the-ankle) amputation is high [24].

The escalating demand for access to care and the direct costs of care for people with diabetic foot complications are comparable to those for cancer [25]. In addition to high treatment costs, the personal burden of the condition is devastating. Lived experience narratives highlight the profound emotional and psychological toll of the condition. Participants in a qualitative study by Lucas [26] described their reaction to the diagnosis as “shock, absolute shock”. The “absolute horror” of feeling “knocked sideways” portrays a sense of “bereavement” and loss of “the life I had”. There is often attribution of blame for developing Charcot foot, directed either at individuals themselves and/or at healthcare professionals [22]. The uncertainty and variability in recovery timelines are a cause of additional frustration [27].

Management often involves prolonged casting, protective footwear and orthotics necessitating frequent healthcare appointments. Daily functioning is often severely disrupted, affecting the ability to walk, drive, work, engage in hobbies and maintain social roles [22]. People living with a Charcot foot often feel “trapped at home” and isolated, and the loss of social life to some feels almost like “identity loss” [26]. Of further concern for the individuals living with CNO is the economic burden, with loss of personal earnings and burden to carers [28]. All of this accounts for a high level of anxiety and depression, emphasising the need for a holistic approach in the management of CNO [29].

### 2.4. Action Required

A recent evaluation of European clinical practice guidelines for the diagnosis and management of the Charcot foot in diabetes raised concern about the limited information and rigour of evidence-based national recommendations, which lack guidance on assessment protocols and treatment pathways [30]. To address this need, the Charcot Guidance Group of the International Working Group on the Diabetic Foot has recently published evidence-based recommendations for the diagnosis and management of CNO, with an emphasis on early recognition, prompt diagnosis, and timely management [1,10].

In this manuscript, we aimed to raise the wider awareness of CNO among healthcare providers by illustrating the presenting signs and symptoms, as well as by providing a structured approach to the assessment and management of the Charcot foot. The narrative has been supported by clinical photographs, imaging studies and decision tools to guide diagnosis and management. We also encourage the use of standardised definitions of the stages of CNO (Diagnosis, Remission and Relapse) to allow comparisons between studies and reporting outcomes [31].

## 3. Presentations and Investigations

### 3.1. Bedside Assessment

“*Always consider active Charcot neuro-osteoarthropathy in a person with diabetes mellitus, neuropathy and intact skin when there are clinical findings of an increase in temperature, oedema, and/or redness of the foot, compared to the contralateral foot*” [1].

This best practice statement, put forward by the International Working Group in the Charcot Guidance document, provides a succinct summary of the presenting signs and symptoms of the active Charcot foot. The Group agrees that the risk of overdiagnosis outweighs the risk of misdiagnosis and delayed institution of treatment. Thus, at present, in cases with a clinically suspected CNO, the person should be treated as the diagnosis has been confirmed until proven otherwise.

**Figure 1:**
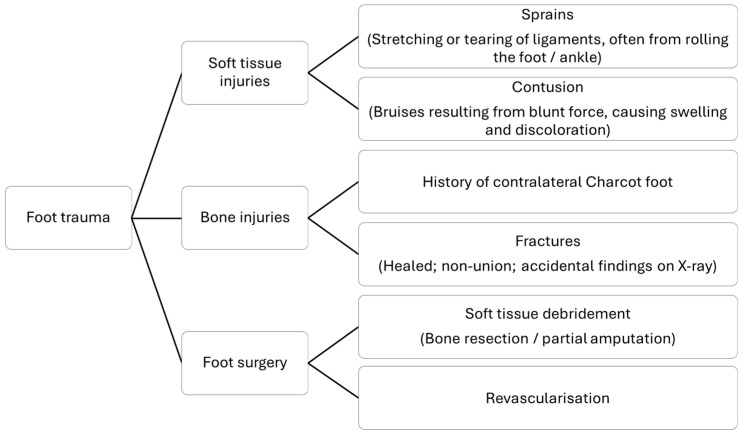
Foot trauma history assessment of the suspected Charcot foot.

The approach to initial assessment should include detailed medical and foot history, including history of foot ulcers and CNO, as well as history of foot trauma (Figure 1).

Standard assessment of neuropathy and peripheral blood supply should be documented. Due to peripheral neuropathy, people’s ability to detect or recall trauma is concealed or even lacking, and some may recall events only when prompted. Lack of pain or discomfort is a frequent finding even in those presenting with significant bone and joint destruction. In some cases, the onset of CNO can resemble that of severe trauma, and it should not be excluded in the absence of a documented medical history of diabetes mellitus [32].

**Figure 2:**
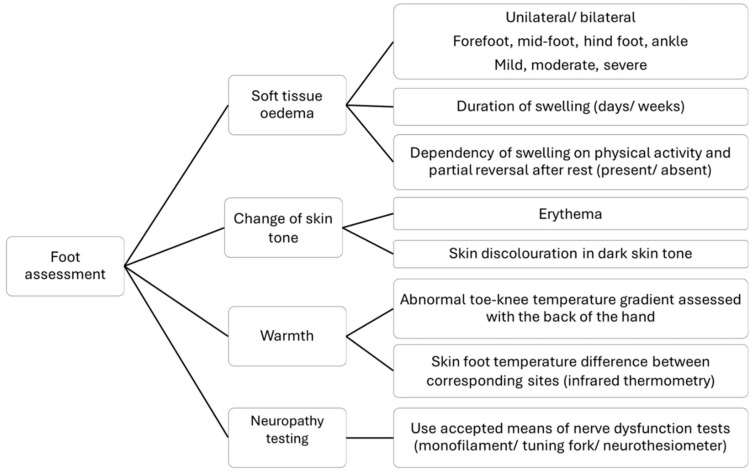
Foot assessment of the suspected Charcot foot.

Foot assessment should include inspection, palpation and temperature testing (Figure 2). Presence of clinical deformity at the onset of CNO should be documented. If the onset of CNO is missed, deformity can be complicated by an ulcer, which sets off a chain of untoward events through infection and risk of amputation [19].

**Figure 3:**
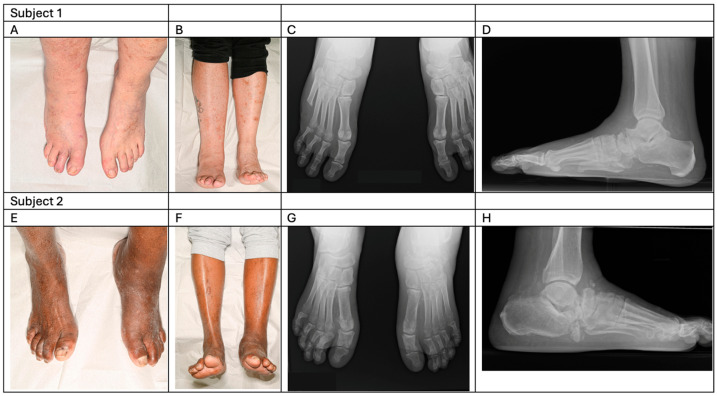
Representative examples of active CNO in diabetes. Subject 1—swelling and erythema of the right foot and leg (**A**,**B**) with talo-navicular bone and joint disruption (**C**,**D**). Subject 2—darkened skin tone and swelling of the left foot and leg (**E**,**F**) with radiological evidence of fragmentation and destruction of the anterior talus, cuboid and navicular bones (**G**,**H**).

The active Charcot foot is most commonly unilateral, and signs and symptoms are limited to the extremity involved (Figure 3A,B,E,F). While the inflammation may present in various sites of the foot and ankle, the mid-foot is the most frequently affected site of bone and joint involvement. In the course of the disease, contralateral foot involvement can develop in almost 34% of the cases. Simultaneous presentation of bilateral active CNO is rare [33]. Nevertheless, the extent of bone destruction can be devastating, necessitating a multidisciplinary approach and referral to specialised diabetic foot care services [34].

Most patients presenting with an active Charcot foot have abnormal findings in nerve function tests (monofilament test, vibration perception threshold and warm and cold sensory thresholds) [35,36]. However, it is important to be aware of an emerging rarer phenotype of the onset of CNO in feet with minimal or no evidence of clinical neuropathy on routine testing [37].

In addition to poor recognition of trauma, pain is another symptom which is often lacking or disregarded at the onset of CNO, and this inevitably accounts for delayed presentation. However, in some presentations, pain can be the leading symptom. It is important to differentiate between mechanical pain (dull ache/discomfort related to episodes of physical activity) from neuropathic pain (random sharp burning night pain, commonly unrelated to physical activity).

Another conundrum is the common understanding that the Charcot foot typically develops in well-perfused feet with bounding pulses. However, recent observational studies have reported active CNO in feet with peripheral arterial disease [38]. This highlights that vascular status may act as a modifier of clinical presentation and outcomes in some individuals with CNO. Further work is needed to clarify whether vascular disease contributes to inter-individual differences in severity and healing trajectories, and whether it may have prognostic value.

Overall, although the Charcot foot is commonly regarded as a unilateral hot, swollen foot in diabetes in feet with neuropathy and preserved blood supply, it is important to be aware of the subtlety of signs and symptoms as well as the wide range of atypical emerging presentations. Early recognition provides a narrow window of opportunity to institute early management [39].

Temperature assessment with skin thermometry is recommended as a first-line assessment of the suspected CNO, as it is the only point-of-care measurement of inflammation. It allows the detection of the temperature difference between both feet. The test should be carried out using a standardised approach to allow for more accurate comparison over time [1]. In the absence of a skin thermometer, the increase in temperature due to the inflammatory process can be detected by palpation and comparison of the distal temperature in the foot to the more proximal temperature in the lower and upper leg (toe to knee temperature).

An increase in temperature of 2 °C or greater on the involved foot compared to the corresponding location on the opposite non-involved foot is often regarded as diagnostic for CNO. However, the Guideline Group questioned the precision of this arbitrary threshold to classify the foot as active when the Charcot foot is ≥2 °C warmer compared to the opposite foot, or inactive foot when the temperature difference between feet is <2 °C. Moreover, a recent systematic review raised concerns regarding the lack of consistency in assessment protocols and disparity within and between practices [40].

Existing protocols are limited to using non-specific thermometers for testing 4 to 8 (non-validated) foot landmarks to detect surface heat, as a surrogate marker of bone damage. Such crude assessment may miss inflammation beyond points of testing and overlook CNO. Conversely, raised foot temperatures are not uncommon in neuropathic feet [41]. All these shortcomings demonstrate an overwhelming need for validated temperature assessment of the suspected Charcot foot, evidenced by imaging studies and robust assessment.

### 3.2. Laboratory Tests

The diagnosis of CNO is primarily based on clinical findings, and there are no established disease markers. The patient should be assessed/screened for uncontrolled/undiagnosed diabetes by evaluating fasting glucose, HbA1c, and/or random glucose levels. Blood tests should also include renal, liver and bone function tests. Inflammatory markers (C-reactive protein, white cell count and erythrocyte sedimentation rate) and uric acid are used as an aid to differentiate CNO from inflammatory foot pathologies.

### 3.3. Differential Diagnosis

The non-specific symptoms of the Charcot foot (erythema or change in skin tone, foot swelling and warmth) may be indistinguishable from other aetiologies of swollen foot in different conditions such as gout, cellulitis or deep vein thrombosis [42].

It is important to differentiate between the red, hot, swollen appearance of the active Charcot foot and the red, hot, swollen cellulitic foot. Cellulitis is more likely in the presence of an ulcer, which may show typical signs of infection. Also, osteomyelitis may be present in such a scenario. The elevation test (lifting the affected extremity above the level of the heart) may be useful to distinguish between erythema (skin tone change) secondary to the inflammation of active Charcot foot and infection. Patients with infection typically do not have resolution of erythema after 5–10 min elevation, whilst erythema consistent with Charcot foot will dissipate after elevation.

Also, C-reactive protein is within the normal range in almost 50% of those with active CNO and only moderately elevated in the remainder [43]. In contrast, a large recent study noted that an ESR of 60 mm/h and a CRP level of 79 mg/L were determined to be the optimal cut-off points for predicting osteomyelitis [44]. White cell count is most commonly normal [43,45].

Gout and deep vein thrombosis may also masquerade as Charcot foot, but can be excluded by measurement of serum uric acid (which is usually raised in gout) and venous duplex ultrasonography (which in CNO will be normal).

It is important to rule out other conditions, such as fasciitis and tendinitis, which may present with similar clinical signs and symptoms but typically do not show osteoarticular abnormalities on radiological imaging [46]. Furthermore, foot injuries (sprains, contusions and minor fractures) often resulting from minor or previously unrecognised trauma in people with diabetes should be readily assessed and timely managed to reduce the risk of progression to CNO bone and changes [47]. 

## 4. Standard and Advanced Imaging Studies

### 4.1. Foot and Ankle Radiographs

Plain X-rays of the foot and ankle should be performed in all persons with diabetes mellitus and suspected active CNO. Ideally, these should be bilateral for comparison purposes and should include weight-bearing (standing) anteroposterior (AP), medial oblique, and lateral views of the foot. The ankle views should include the AP, mortise, and lateral projections [1]. Radiological findings should be related to clinical findings. The forefoot, mid-foot and hind foot should be closely assessed for early signs of soft tissue swelling, joint effusion and osteolysis (regional osteopenia, cortical erosions, fractures and fragmentation, calcic debris, joint subluxation or dislocations).

Previously, the rapid bone and joint destruction noted in individuals with CNO has often been referred to as “osteoarthritis with a vengeance” [48]. Evidence from a bone specimen study has indicated a marked difference in the density of sympathetic nerve fibres, which was reduced in samples from CNO compared with samples from osteoarthritic bone [49].

Several systems have been developed as means of classifying CNO, with emphasis on the anatomical zones of involvement or natural history and disease progression [50]. The evolution of the Charcot foot was documented by Eichenholtz in 1966. In his monograph “Charcot joints”, he documented 68 presentations of Charcot joints in several conditions, including diabetes (*n* = 12), syphilis (*n* = 34), alcoholism (*n* = 4), syringomyelia (*n* = 3) and leprosy (*n* = 1) [51]. Based on the X-ray findings and foot presentation, Eichenholtz delineated three stages in the natural history of the condition: development, coalescence and reconstruction, and reconstitution (Table 1) [52].

Sanders and Frykberg’s classification describes five patterns of bone and joint involvement (Table 2) [52]. Other anatomical classification systems have been described, including the Brodsky classification with later modification by Trepman, which groups disease more broadly by anatomical regions [53].

The Charcot disease in the UK audit reported the mid-foot and hind foot as the most common presentations based on the analysis of 288 new cases of CNO registered within a 20-month period [54]. In addition to structural involvement and extent of damage, radiographic alignment measurements are useful means of grading foot and ankle deformity at presentation and its progression on follow up [55,56,57]. In the longer term, these radiographic-based measures of alignment are useful predictors of ulcer formation in feet with Charcot deformity [58].

Recently, there has been increased recognition of the prodromal stage of CNO (X-ray negative), when the foot is inflamed but skeletal changes are minimal or lacking. The approach to advanced imaging of the suspected CNO with normal X-rays has been directed by the Charcot Guidance Group [1]. Magnetic resonance imaging (MRI) is recommended as first line modality in the assessment of the suspected active CNO when plain X-rays are normal. If MRI is contraindicated or access to this modality is limited, a nuclear imaging scan (scintigraphy), computed tomography (CT) scan or Single Photon Emission Computed Tomography/Computed Tomography (SPECT-CT) is suggested as next-line investigation. More recently, initial experience with the use of dual-energy CT has been reported [59]. A further technique, which has been piloted in the assessment of CNO, is F-18-fluoro-2-deoxyglucose-positron emission tomography computed tomography. (F-18 FDG PET/CT) [60]. However, this modality should be reserved as a second-line investigation, predominantly in diagnostically challenging cases, as current evidence regarding its usefulness is limited.

### 4.2. Magnetic Resonance Imaging

Magnetic resonance imaging is the most studied modality in the assessment of the suspected CNO. It can detect bone and joint abnormalities and signs of inflammation and can support a decision to diagnose or exclude the disease. Key pathological findings include soft tissue swelling and fascial oedema, joint effusion and tenosynovitis, bone marrow oedema ± cortical bone erosions or ±subchondral fractures, bone fragments, subluxations/dislocations. A list of findings is documented in Table 1 of the International Guideline Document [1].

**Figure 4:**
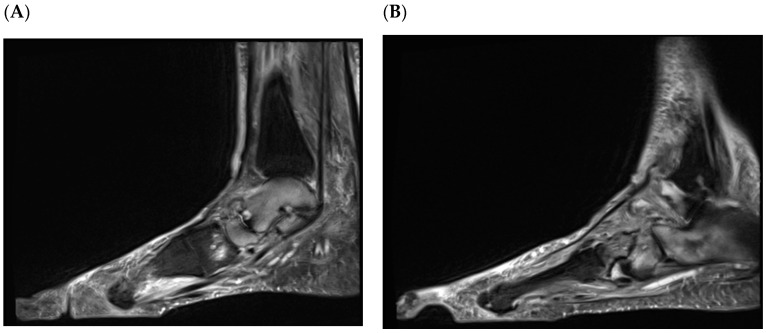
MRI scan of Subject 1 shows, in addition to talo-navicular bone and joint disruption (**A**), extensive bone marrow oedema in the calcaneocuboid region (**B**), which appears non-affected on X-ray (Figure 3D).

It is important to be aware of the poor performance of X-rays in the assessment of the hot, swollen foot in diabetes. A recent agreement study reported that X-ray underscored MRI in grading fractures in the metatarsals (*p* = 0.05) and tarsals (*p* < 0.001), and reported as normal 79% of the bones with bone marrow oedema [61] (Figure 4). This should be considered when confirming or refuting the diagnosis of active CNO.

A further differential diagnosis which should be considered in people living with diabetes, presenting with hot swollen foot and normal radiograph is complex regional pain syndrome I (CRPS I), previously known as Sudeck’s disease stage 1. The clinical features and MRI findings in a series of cases presenting CRPS I and CNO have been summarised in a recent report, including patients’ photographs and imaging studies [62].

### 4.3. Bone Scintigraphy and SPECT-CT

A nuclear imaging scan (bone scintigraphy) uses a radioactive tracer, technetium-99m methylene diphosphonate (^99m^Tc-MDP), which is absorbed by areas of bone with increased activity. This modality detects inflammation and bone changes in CNO. Foot and ankle images show asymmetry and increased activity of the tracer in the blood phase, and in the early blood pool and bony phases of the scan. The more advanced technique, such as SPECT/CT, provides images that help determine the exact location of the bony pathology [63].

In a cross-sectional series of people with diabetes presenting with suspected Charcot foot, SPECT/CT demonstrated that the X-ray negative (Stage 0) Charcot foot had three distinct bone phenotypes: (1) fractures detected on CT associated with focal uptake of tracer on SPECT, (2) bony abnormalities apart from fracture on CT with focal uptake of tracer on SPECT, and (3) normal CT but focal bony uptake of tracer on SPECT [63].

### 4.4. Computed Tomography

Computed tomography is a high-resolution imaging modality used in the assessment of osseous structures. In addition to visualising fractures, fragmentation and dislocations in the active stage of CNO, the three-dimensional CT reconstructions are essential in assessing the extent of osseous involvement, particularly in cases with deformity before surgical decision-making and planning [64]. Dual-energy CT (DECT) is an emerging technology that can provide virtual non-calcium images, allowing assessment of bone marrow oedema in the suspected active CNO. Recent work has shown that mid-foot bone marrow oedema identified on DECT correlates with clinically active disease, suggesting that DECT may be a useful alternative when MRI is unavailable or unsuitable [59]. 

### 4.5. Clinical Suspicion of CNO Is Fundamental for Early Diagnosis

A high index of suspicion is the most useful tool to diagnose CNO. It is to consider the onset even when signs and symptoms deviate from classical presentation (no previous record of diabetes, no signs of neuropathy on routine assessment, insignificant past foot history, foot pain or signs of peripheral vascular disease). Although the phenotypes described above are rare, these atypical presentations might preclude early diagnosis and, consequently, delay management.

It is now well accepted that identification of CNO at the prodromal stage, when the foot is inflamed but skeletal changes are minimal or lacking, provides an emerging window of opportunity to prevent bone and joint destruction [39]. Earlier diagnosis will lead to lesser degrees of deformity, reduce the risk of foot ulceration and may also enhance quality of life [12]. Evidence from retrospective studies suggests that feet diagnosed and managed early are more likely to heal without deformity [63,65,66]. In contrast, a delayed diagnosis of 8 weeks or more is associated with a 5-fold risk of adverse outcomes [67].

The best practice recommendation is to initiate treatment while waiting for confirmatory imaging studies, as the risk of treatment outweighs the risk of foot collapse and adverse events [1].

## 5. Current Approach to Management

### 5.1. Offloading in Active CNO

Present management includes offloading the foot in a below-knee cast. A non-removable knee-high total contact cast is the preferred offloading treatment of active CNO [1]. If this modality is not available or unacceptable to the patient, the second choice should be a knee-high walker boot rendered non-removable. A removable knee-high device worn at all times may be considered when non-removable options are not feasible. Assistive devices (crutches, mobility scooters or Zimmer frames) should be considered in all cases to facilitate mobility and limit weight-bearing on the affected limb.

When managing people in long-term offloading with a non-removable total contact cast (TCC), one should take an individualised approach with consideration of both personal and societal factors (Table 3). This will ensure holistic and successful treatment, which often involves a multidisciplinary approach. Providing sufficient information about the benefits of TCC is essential for the person with diabetes to make an informed choice.

Offloading the limb can take up to several months [54]. Safe delivery of TCC therapy for individuals with neuropathy and diabetes relies on strict adherence to application by a trained healthcare professional and monitoring, and rigorous patient education and follow-up. There should be a safety network for regular cast checks, follow-up replacement and out-of-hours emergency care.

**Figure 5:**
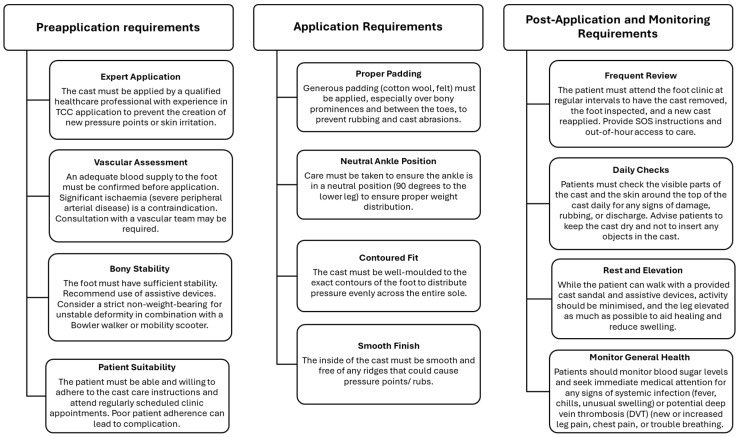
Safe delivery of total contact cast (TCC) therapy for individuals with neuropathy and diabetes.

Both the patient and healthcare providers need to understand the associated risks of managing a neuropathic limb in a closed, non-removable cast [68]. The risk of TCC-related complications is up to 10% [68]. However, most of these adverse events (ingrown toenails, dry skin, cast abrasions, foot ulcers) can be resolved following cast change or cast removal. In addition, the Charcot Guidance Group recognises that prolonged use of knee-high immobilisation may be accompanied by broader consequences, including muscle weakness, risk of falls and musculoskeletal strain [1]. Awareness of these potential effects is important when planning care, emphasising the need to balance effective protection of the foot with preservation of overall function wherever this can be achieved safely. Figure 5 provides a structured approach to safe delivery of TCC therapy for individuals with neuropathy and diabetes.

### 5.2. Remission of Active CNO

Determining remission of active CNO remains challenging, as no single clinical measure or investigation can reliably confirm healing in isolation [1]. Current practice therefore relies on a combination of clinical assessment, including reduction in swelling, erythema and skin temperature difference between feet, alongside serial imaging findings.

Plain radiographs remain the most widely used imaging modality for monitoring CNO, reflecting their accessibility and routine use in clinical practice. However, interpretation of radiographs when assessing remission can be difficult, as radiographic changes may lag clinical improvement. Furthermore, structural changes may continue to evolve over time despite apparent clinical remission and resolution of inflammatory features [39,56]. This can result in discordance between different indicators of disease activity, with clinical improvement and temperature normalisation not always aligning with imaging findings.

Magnetic resonance imaging abnormalities, such as bone marrow oedema, have been reported to persist despite apparent clinical improvement, complicating interpretation during follow-up [69,70,71]. Consequently, the interpretation and relative weighting of serial imaging findings when confirming remission remains uncertain, and their use may influence the duration of immobilisation and timing of transition to footwear. Decisions regarding cessation of offloading and progression to rehabilitation are individualised and typically informed by integrated clinical assessment, often within a multidisciplinary care setting.

Even after apparent remission, individuals with a history of CNO remain at high risk of reactivation, progressive deformity and ulcer-related complications [1,24,72]. Continued surveillance, ongoing access to specialist foot services and optimisation of protective strategies are therefore important aspects of care beyond the acute episode. Future refinements in risk stratification may help tailor the intensity of follow-up according to individual risk.

### 5.3. Conservative and Surgical Approach–Consideration and Planning

Patients with stable deformity and low risk of ulceration, or no deformity, should be managed with below-knee casting until resolution of inflammation (reduction in swelling and warmth of the foot) and bone healing. A transition from a non-removable to a removable cast with gradual rehabilitation into custom-made insoles and custom-made footwear is recommended to reduce the risk of ulceration and reactivation of CNO.

However, there is currently no standardised approach to the timing or process of transition from immobilisation during active CNO to custom-made footwear. Evidence guiding this transition phase remains limited, and data on rates and predictors of reactivation or recurrence are sparse. Observational studies suggest that, despite effective offloading and high adherence to custom-made footwear, ulcer recurrence remains common in people with CNO [72]. This underscores the need for further research into optimal transition strategies and long-term management.

Patients presenting with unstable Charcot deformity should be assessed for their suitability for internal/external surgical reconstruction. The decision should be based on the experience and availability of care, as well as access to a multidisciplinary team for long-term follow-up [1,73,74]. Some patients with stable deformity and high risk of ulceration may benefit from exostectomy, which is a less aggressive form of surgery aimed at local reduction of pressure at a plantar or medial bony prominence [75].

### 5.4. Is There a Role in Pharmacotherapy of the Charcot Foot?

The efficacy and benefits of medical therapies adjunctive to offloading therapy for the active Charcot foot have been a topic of significant interest and investigation over the last 25 years. Current international guidelines do not recommend the routine use of pharmacological therapy as an adjunct to offloading in active CNO [1].

Initially, the usefulness of antiresorptive agents was tested, and this included clinical trials with intravenous (pamidronate or zolenronate) or oral (alendronate) bisphosphonates or subcutaneous administration of calcitonin [76,77,78,79]. However, evidence to support their use was found weak [80], and neither therapy is recommended for clinical use.

An alternative approach aimed at correcting the imbalance between the extensive bone resorption and the impaired bone healing of the active CNO investigated the use of the anabolic agent, recombinant human parathyroid hormone [81]. Although the median time to remission was 4 weeks shorter in the active compared with the placebo group, this difference did not reach statistical significance, and therefore this therapy is also not recommended.

The perpetual role of inflammation as a driver of pathological osteolysis of the active Charcot foot has been demonstrated in both clinical and experimental research [82,83]. Das et al. investigated the efficacy of methylprednisolone compared to placebo in patients with active CNO, using offloading in a TCC as the primary treatment modality in a randomised, placebo-controlled trial of 36 participants [84]. Despite its anti-inflammatory effect, therapy with methylprednisolone resulted in longer time to remission compared with controls. Furthermore, this therapy did not provide any improvement in long-term clinical outcomes, including the incidence of deformities, ulcers or amputations [85]. Thus, it is also not recommended in the management of CNO.

More recently, using advances in bone biology, studies on the pathogenesis of CNO have identified highly activated osteoclasts as key drivers of the extensive bone destruction of the active Charcot foot [86,87]. Osteoclastic precursors express receptor activator of nuclear factor kappa-B (RANK) and circulate in the monocytic fraction of blood and can be found at sites of inflamed joints and injured bones [88]. Osteoclastic activity is mediated by the receptor activator of nuclear factor kappa-B Ligand (RANKL), which is released by osteoblasts. Recently, there has been significant interest in the potential benefit of denosumab, a fully human monoclonal anti-RANKL antibody, in the management of CNO [89]. This agent has a high affinity for RANKL and prevents it from interacting with its receptor, RANK, by blocking the binding between the two on the surface of osteoclast precursors [90].

The effectiveness of this novel biologic agent in CNO was recently evaluated. Busch-Westbroek et al. found that patients treated with denosumab had a significantly shorter time to fracture healing and time to clinical cessation of offloading therapy when compared to a historical control group of patients [91]. Two further observational open-label studies in subjects with active CNO reported that this therapy was well tolerated and was not associated with adverse events [92,93]. However, a recent multi-centre open-label phase 2 randomised controlled trial indicated that therapy with denosumab in CNO did not reduce time to cessation of offloading between active and placebo therapy despite the protective effect on calcaneal stiffness index noted in the active group [94]. Thus, the benefit of denosumab as adjunctive therapy in CNO remains not fully known. It is expected that ongoing research may provide more clarity on the potential usefulness of this novel therapeutic approach [95,96].

## 6. Conclusions

In 2025, we marked the 200th anniversary of the birth of Jean-Martin Charcot (1825–1893), the French neurologist from the Salpêtrière Hospital in Paris, whose innovative approach in clinical studies, research, and education has left a lasting impact on medicine [97].

On the bicentennial anniversary, Jean-Martin Charcot’s legacy remains central to modern diabetic foot care. His extraordinary commitment to close clinical observation continues to guide clinicians in recognising the earliest signs and symptoms of CNO—often subtle, easily missed, yet critical to detect. Charcot’s emphasis on correlating clinical findings with detailed imaging analysis underpins today’s diagnostic pathways, enabling earlier identification of structural changes before irreversible deformity occurs.

Most importantly, his principles support prompt, targeted management that reduces complications, preserves limb function, improves long-term outcomes and quality of life. Charcot’s influence endures not as a historical tribute but as a living framework that shapes contemporary best practice and transforms patient care.

## Figures and Tables

**Table 1:** Eichenholtz’s description of the natural course of the Charcot foot (radiological features and foot presentation).

Stages	Radiological Features	Foot Presentation (Appearance)
Stages of development	Debris Fragmentation Disruption Dislocation	Redness Swelling Warmth Bounding pulses
Stages of coalescence	Sclerosis Absorption of fine debris Fusion of most large fragments	No redness Reduced swelling No warmth
Stage of reconstruction and reconstitution	Lessened sclerosis Rounding of major fragments Attempts at reformation of joint architecture	Ultimate foot deformity Rocker bottom deformity Medial convexity Ankle subluxation

**Table 2:** Sanders and Frykberg’s anatomical classification of foot and ankle radiographs.

Patterns of Presentation	Bone and Joint Involvement
Pattern I	Metatarsal/phalangeal joints
Pattern II	Metatarsal/tarsal joints (Lisfranc joints)
Pattern III	Midtarsal joints (Chopart joints)
Pattern IV	Ankle and subtalar joint
Pattern V	Calcaneum

**Table 3:** Personal and societal factors influencing long-term non-removable offloading in active Charcot neuro-osteoarthropathy.

Personal Factors	Societal Factors
**Psychological Impact** Anxiety related to casting, low mood, distress, sleep disturbance, and reduced self-esteem may affect tolerance of prolonged offloading.	**Social isolation** Physical restrictions and visibility of the cast may reduce participation in social, recreational, or occupational activities.
**Mobility and independence** Reduced mobility and loss of independence can interfere with activities of daily living, including personal care and sleep.	**Employment and finances** Prolonged offloading may affect employment and income. Travel costs and time off work for appointments can add financial burden.
**Adherence and preference** Engagement with treatment and attendance at follow-up are essential. Cast tampering or removal may compromise treatment.	**Support systems** Access to family or community support is important. Lack of in-home support may reduce adherence and safety.
**Body image and aesthetics** The visible appearance of a cast may affect self-image and willingness to engage in social activities.	**Healthcare resources and skills** Availability of trained clinicians and local expertise influences access to safe and timely non-removable offloading.
**Comorbidities** Conditions such as balance impairment, musculoskeletal disease, previous amputation, or visual impairment may affect the ability to manage non-removable offloading.	**Accessibility and environment** Physical barriers such as stairs, housing layout, and transport availability may limit mobility and access to care.

## Data Availability

No new data were created or analysed in this study.

## Abbreviations

The following abbreviations are used in this manuscript:

99mTc-MDP	Technetium-99m methylene diphosphonate
AP	Anteroposterior
CNO	Charcot neuro-osteoarthropathy
CRPS I	Complex regional pain syndrome I
CRP	C-reactive protein
CT	Computed tomography
ESR	Erythrocyte sedimentation rate
F-18 FDG PET/CT	F-18-fluoro-2-deoxyglucose-positron emission tomography computed tomography
HbA1c	Glycated haemoglobin A1c
HIV	Human immunodeficiency virus
ICD-10	International Classification of Diseases, 10th Revision
MRI	Magnetic resonance imaging
RANK	Receptor activator of nuclear factor kappa-B
RANKL	Receptor activator of nuclear factor kappa-B ligand
SPECT-CT	Single photon emission computed tomography–CT
TCC	Total contact cast
